# APOBEC-mediated mutagenesis in urothelial carcinoma is associated with improved survival, mutations in DNA damage response genes, and immune response

**DOI:** 10.18632/oncotarget.23344

**Published:** 2017-12-16

**Authors:** Alexander P. Glaser, Damiano Fantini, Yiduo Wang, Yanni Yu, Kalen J. Rimar, Joseph R. Podojil, Stephen D. Miller, Joshua J. Meeks

**Affiliations:** ^1^ Department of Urology, Northwestern University, Feinberg School of Medicine, Chicago, IL, USA; ^2^ Robert H. Lurie Comprehensive Cancer Center, Northwestern University, Chicago, IL, USA; ^3^ Interdepartmental Immunobiology Center, Department of Microbiology-Immunology, Northwestern University, Chicago, IL, USA

**Keywords:** urinary bladder neoplasms, APOBEC deaminases, mutagenesis, DNA damage, interferon

## Abstract

APOBEC enzymes are responsible for a mutation signature (TCW>T/G) implicated in a wide variety of tumors. We explore the APOBEC mutational signature in bladder cancer and the relationship with specific mutations, molecular subtype, gene expression, and survival using sequencing data from The Cancer Genome Atlas (*n* = 395), Beijing Genomics Institute (*n* = 99), and Cancer Cell Line Encyclopedia. Tumors were split into “APOBEC-high” and “APOBEC-low” based on APOBEC enrichment. Patients with APOBEC-high tumors have better overall survival compared to those with APOBEC-low tumors (38.2 vs. 18.5 months, *p* = 0.005). APOBEC-high tumors are more likely to have mutations in DNA damage response genes (*TP53, ATR, BRCA2*) and chromatin regulatory genes (*ARID1A, MLL, MLL3*), while APOBEC-low tumors are more likely to have mutations in *FGFR3* and *KRAS*. *APOBEC3A* and *APOBEC3B* expression correlates with mutation burden, regardless of bladder tumor molecular subtype. APOBEC mutagenesis is associated with increased expression of immune signatures, including interferon signaling, and expression of *APOBEC3B* is increased after stimulation of APOBEC-high bladder cancer cell lines with IFNγ. In summary, APOBEC-high tumors are more likely to have mutations in DNA damage response and chromatin regulatory genes, potentially providing more substrate for APOBEC enzymes, leading to a hypermutational phenotype and the subsequent enhanced immune response.

## INTRODUCTION

Urothelial carcinoma has one of the highest mutation rates of any sequenced cancer to date along with lung cancer and melanoma [1]. High-throughput next generation sequencing analyses such as The Cancer Genome Atlas (TCGA) and others have identified a mutational signature characterized by a TCW>T/C mutation thought to be attributable to the apolipoprotein B mRNA editing catalytic polypeptide-like (APOBEC) family of enzymes [1–3]. This mutational pattern is the predominant pattern in muscle-invasive bladder cancer (approximately 80% of bladder tumors in the TCGA have an APOBEC mutation signature) and is also frequently found in breast, cervical, head and neck, and lung cancers [1, 3–5].

The APOBEC family consists of 11 members, including *AID*, *APOBEC1*, *APOBEC2*, *APOBEC3A*, *APOBEC3B*, *APOBEC3C*, *APOBEC3D*, *APOBEC3F*, *APOBEC3G*, *APOBEC3H*, and *APOBEC4*. These enzymes function as cytosine deaminases and are involved in C>U deamination in single-stranded DNA (ssDNA), and likely function physiologically in antiretroviral defense [6–9]. However, in tumor cells, these enzymes are likely responsible for hypermutation at cytosine bases in exposed ssDNA [10]. The APOBEC3 family, and particularly *APOBEC3A* and *APOBEC3B* [6, 11–14], are the predominant APOBEC enzymes theorized to contribute to cancer mutagenesis.

Several studies have linked *APOBEC3B* expression with mutagenesis [5, 14, 15], but its expression alone does not fully explain this mutational signature, and *APOBEC3A* may also play a significant role [11]. Regulation of APOBEC enzymes remains unclear, but expression of *APOBEC3A* and *APOBEC3B* can be induced in bladder cancer cell lines by the DNA-damaging agent bleomycin, as well as by an interferon response [4]. In TCGA breast and bladder cancers, DNA replication stress and mutations in DNA repair genes have been linked to APOBEC-mediated mutagenesis [4, 16], potentially due to increased availability of ssDNA substrate for enzymatic deamination [17, 18]. Furthermore, a mutation in *TP53* or other DNA damage response genes may be a prerequisite for cancer cells to survive in the setting of APOBEC-driven kataegis [15].

In this study, we investigate the APOBEC mutational signature in the TCGA, Beijing Genomics Institute (BGI), and Cancer Cell Line Encyclopedia (CCLE) bladder cancer datasets and its relationship with specific mutations, molecular subtype, gene expression, and survival. We hypothesized that tumors with high levels of APOBEC-mediated mutagenesis would be enriched for mutations in DNA damage response genes and express genes related to activation of the immune system at higher levels, while tumors with low levels of APOBEC-mediated mutagenesis may have enrichments for oncogenes. Thus, APOBEC activity may link DNA damage and immune response in urothelial carcinoma.

## RESULTS

### APOBEC mutagenesis in bladder cancer

**Figure 1:**
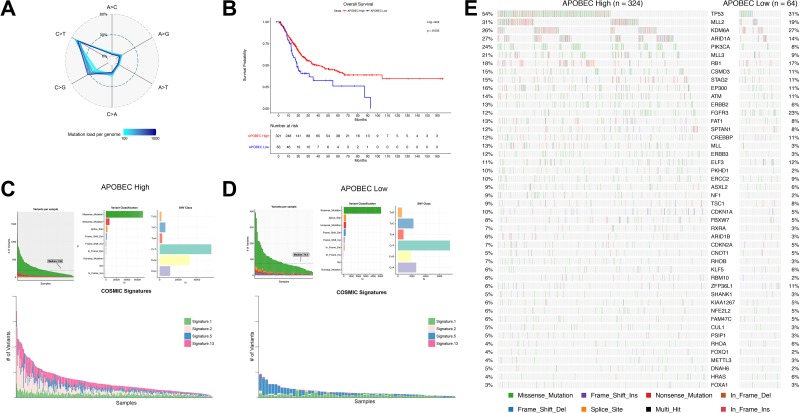
APOBEC-mediated mutagenesis in the TCGA bladder cancer cohort (*n* = 388) (**A**) Percentage of single nucleotide variations (SNVs) as a function of mutation load. Genomes were binned in groups of 20 samples according to mutation load. (**B**) Kaplan-Meier survival curve of APOBEC-high and APOBEC-low bladder tumors. (**C**) Summary of mutagenesis in APOBEC-high tumors, including number of variants per sample, variant classification, class of SNV, and contribution of COSMIC signatures 1, 2, 5, and 13 in each sample. (**D**) Summary of mutagenesis in APOBEC-low tumors, including number of variants per sample, variant classification, class of SNV, and contribution of COSMIC signatures 1, 2, 5, and 13 in each sample. (**E**) Oncoplot of the top genes commonly mutated in the TCGA bladder cancer cohort in APOBEC-high and APOBEC-low tumors. TCGA, The Cancer Genome Atlas; COSMIC, catalogue of somatic mutations in cancer.

To understand factors associated with APOBEC mutagenesis, we first evaluated the association of APOBEC signature with mutation burden in the TCGA cohort. When compared across all patients, we found the frequency of the nucleotide conversion C>G mutations is directly related to total mutation burden in bladder cancer (Figure 1A). Many of these mutations are a specific contextual TCW>T/G mutation attributed to the APOBEC family of enzymes. Of 388 tumors in the provisional TCGA bladder urothelial carcinoma dataset, 324 are enriched for APOBEC mutagenesis (“APOBEC-high”) vs. 64 with low or no enrichment (“ABPOEC-low”) [19, 20]. When stratified by APOBEC enrichment, APOBEC-high tumors were associated with almost a two-fold increase in overall survival compared to APOBEC-low tumors (median overall survival 38.2 vs 18.5 months, *p* = 0.0050, Figure 1B). TCGA APOBEC-high tumors have a higher number of variants per sample, a higher proportion of C>T and C>G mutations, and a higher proportion of catalogue of somatic mutations in cancer (COSMIC) signatures 2 and 13, as expected (Figure 1C–1D). APOBEC-low tumors were mainly comprised of signatures 1 and 5.

**Figure 2:**
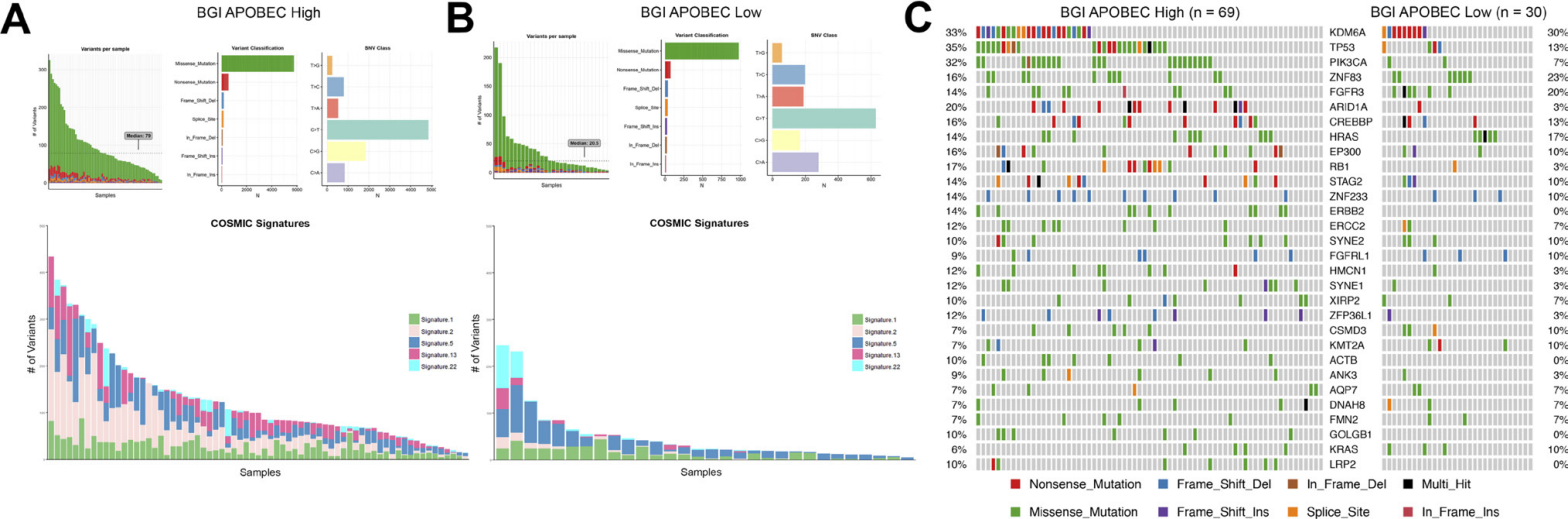
APOBEC-mediated mutagenesis in the BGI bladder cancer cohort (*n* = 99) (**A**) Summary of mutagenesis in APOBEC-high tumors, including number of variants per sample, variant classification, class of SNV, and contribution of COSMIC signatures 1, 2, 5, 13, and 22 in each sample. (**B**) Summary of mutagenesis in APOBEC-low tumors, including number of variants per sample, variant classification, class of SNV, and contribution of COSMIC signatures 1, 2, 5, 13, and 22 in each sample. (**C**) Oncoplot of the top genes commonly mutated in the BGI bladder cancer cohort in APOBEC-high and APOBEC-low tumors. BGI, Beijing Genomics Institute; COSMIC, catalogue of somatic mutations in cancer.

To confirm our findings, we investigated APOBEC signature and mutagenesis in the BGI bladder cancer cohort. BGI APOBEC-high tumors were also found to have higher mutation burden and a higher proportion of APOBEC COSMIC signatures 2 and 13 (Figure 2A and 2B). COSMIC signature 22 was also represented in some samples of both the BGI APOBEC-high and APOBEC-low cohorts, with signature 22 found at higher mutation burden (Figure 2A and 2B; cyan color).

We then investigated the clinical and pathologic features associated with APOBEC enrichment. APOBEC-low tumors are more likely to be low-grade in the TCGA cohort (17% vs 3%, *p <* 0.0001; Supplementary Table 1A). A higher proportion of APOBEC-low tumors from the BGI dataset were Grade 1/3 (50% vs 38%), but this was not statistically significant (Supplementary Table 1B). A higher frequency of Asian patients were APOBEC-low vs. APOBEC-high in the TCGA cohort (26% vs 7%), and *APOBEC3B* was expressed at a lower level in Asian patients vs. non-Asian ethnicity (*p* < 0.0001, Supplementary Figure 2). In addition, a higher proportion of patients in the BGI dataset were APOBEC-low (30%), compared to the TCGA dataset (16%; *p* = 0.0027), confirming the association of a lower level of APOBEC enrichment with Asian ethnicity. APOBEC-high and -low tumors were otherwise were similar in stage, subtype, gender, and smoking history in both cohorts (Supplementary Table 1A–1B).

### Differential mutations in APOBEC-high and APOBEC-low tumors

**Figure 3:**
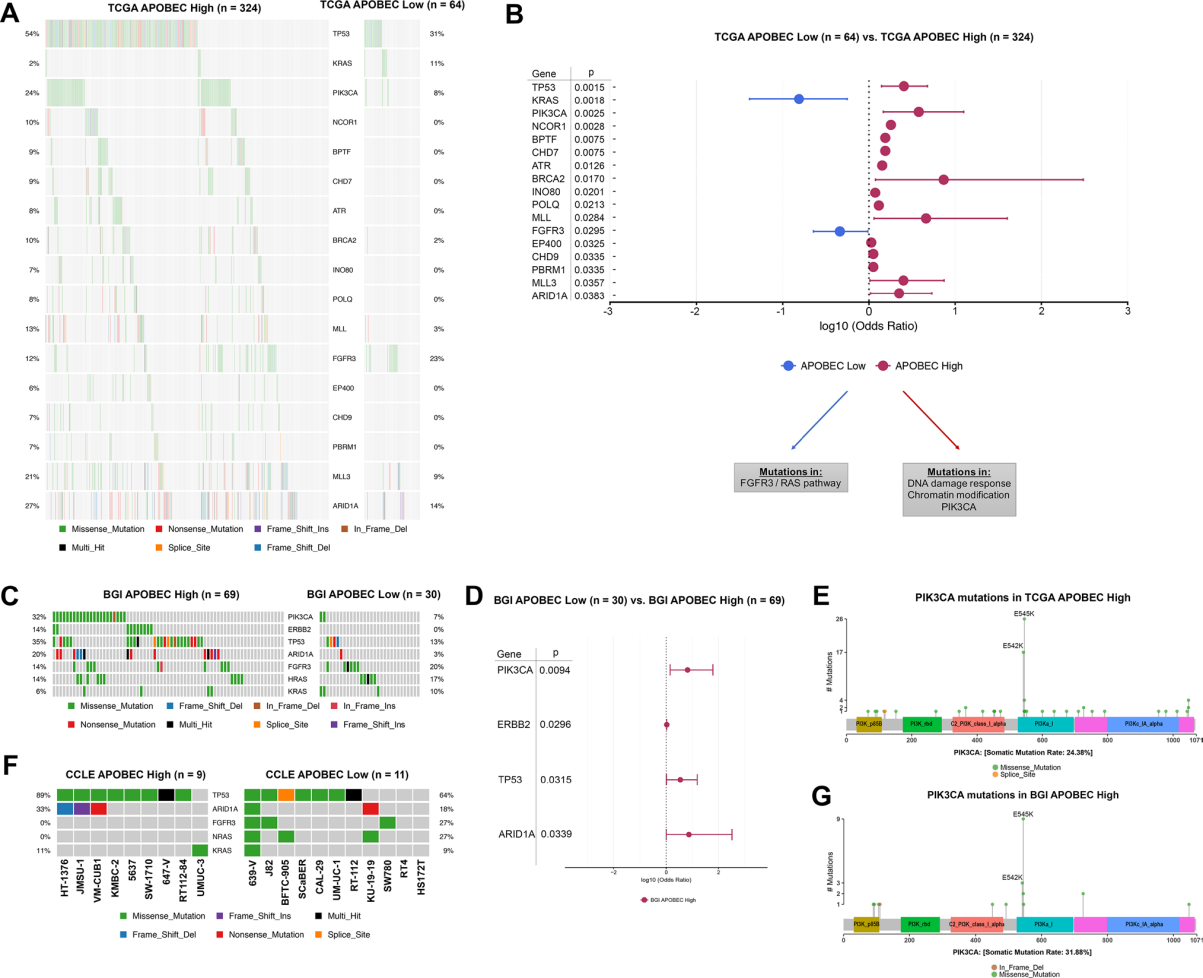
Differentially mutated genes in APOBEC-high and APOBEC-low tumors (**A**) Oncoplot of genes significantly differentially mutated in TCGA APOBEC-high and APOBEC-low tumors. (**B**) Forestplot of differentially mutated genes in TCGA APOBEC-high and APOBEC-low tumors with log10 odds ratio, 95% confidence intervals, and adjusted *p*-value. (**C**) Oncoplot of significantly differentially mutated genes (*PIK3CA, ERBB2, TP53, ARID1A*) as well as *FGFR3*/RAS genes in BGI APOBEC-high and APOBEC-low tumors. (**D**) Forestplot of differentially mutated genes in BGI APOBEC-high and APOBEC-low tumors with log10 odds ratio, 95% confidence intervals, and adjusted *p*-value. (**E**) Lollipop plot of *PIK3CA* mutations in TCGA APOBEC-high tumors. The majority of mutations in *PIK3CA* in APOBEC-high tumors are in the helical domain at TCW motifs 542 and 545. (**F**) Oncoplot of *TP53*, *ARID1A, FGFR3, NRAS*, and *HRAS* in bladder cancer cell lines. (**G**) Lollipop plot of *PIK3CA* mutations in BGI APOBEC-high tumors. Again the majority of mutations in *PIK3CA* in APOBEC-high tumors are in the helical domain at TCW motifs 542 and 545. TCGA, The Cancer Genome Atlas. BGI, Beijing Genomics Institute

To determine what somatic mutations were associated with APOBEC mutagenesis in bladder tumors, we next compared mutated genes between APOBEC-high and APOBEC-low tumors. After correction for multiple comparisons, TCGA APOBEC-high tumors were more likely to have mutations in *TP53*, *PIK3CA*, *ATR*, *BRCA2*, *MLL*, *MLL3*, and *ARID1A* while TCGA APOBEC-low tumors were more likely to have mutations in *KRAS* and *FGFR3* (Figure 3A–3B; Supplementary Table 2). Functional annotation of differentially mutated genes demonstrates that APOBEC-high tumors are enriched for mutations in DNA damage repair genes and chromatin modification genes (Supplementary Table 3).

We next confirmed these analyses by evaluation of the mutation profile of APOBEC-high and APOBEC-low tumors in the smaller BGI cohort. The most frequent mutations in the BGI APOBEC-high tumors were *TP53* (35%), *KDM6A* (33%), *PIK3CA* (32%), and *ARID1A* (20%), whereas the most frequent mutations in the APOBEC-low tumors were *KDM6A* (30%), *ZNF83* (23%), *FGFR3* (20%), and *HRAS* (17%). BGI APOBEC-high tumors were significantly more likely to have mutations in *PIK3CA*, *ERBB2*, *TP53*, and *ARID1A* (Figure 3C; Supplementary Table 4).

Non-synonymous mutations in *KRAS* and *FGFR3* are mutually exclusive in both the BGI and TCGA cohorts (Figure 3A and 3C). Mutations in PIK3CA occurred primarily at E542K and E545K in both the TCGA and BGI cohorts, which are APOBEC TCW motifs (Figure 3E and 3G), suggesting these mutations may be a result of APOBEC-mediated mutagenesis rather than a driver of the APOBEC mutational signature.

Finally, we confirmed the mutational patterns by analysis of multiple CCLE bladder cancer cell lines. Of 20 bladder cancer cell lines, 9 were enriched for APOBEC mutagenesis, and had frequent mutations in *TP53* (89%) and *ARID1A* (33%), while 11 cell lines were not enriched for APOBEC mutagenesis and had frequent mutations in *FGFR3* (27%) and *NRAS* (27%) (Figure 3F).

### Expression of APOBEC3 correlates with mutational burden

**Figure 4:**
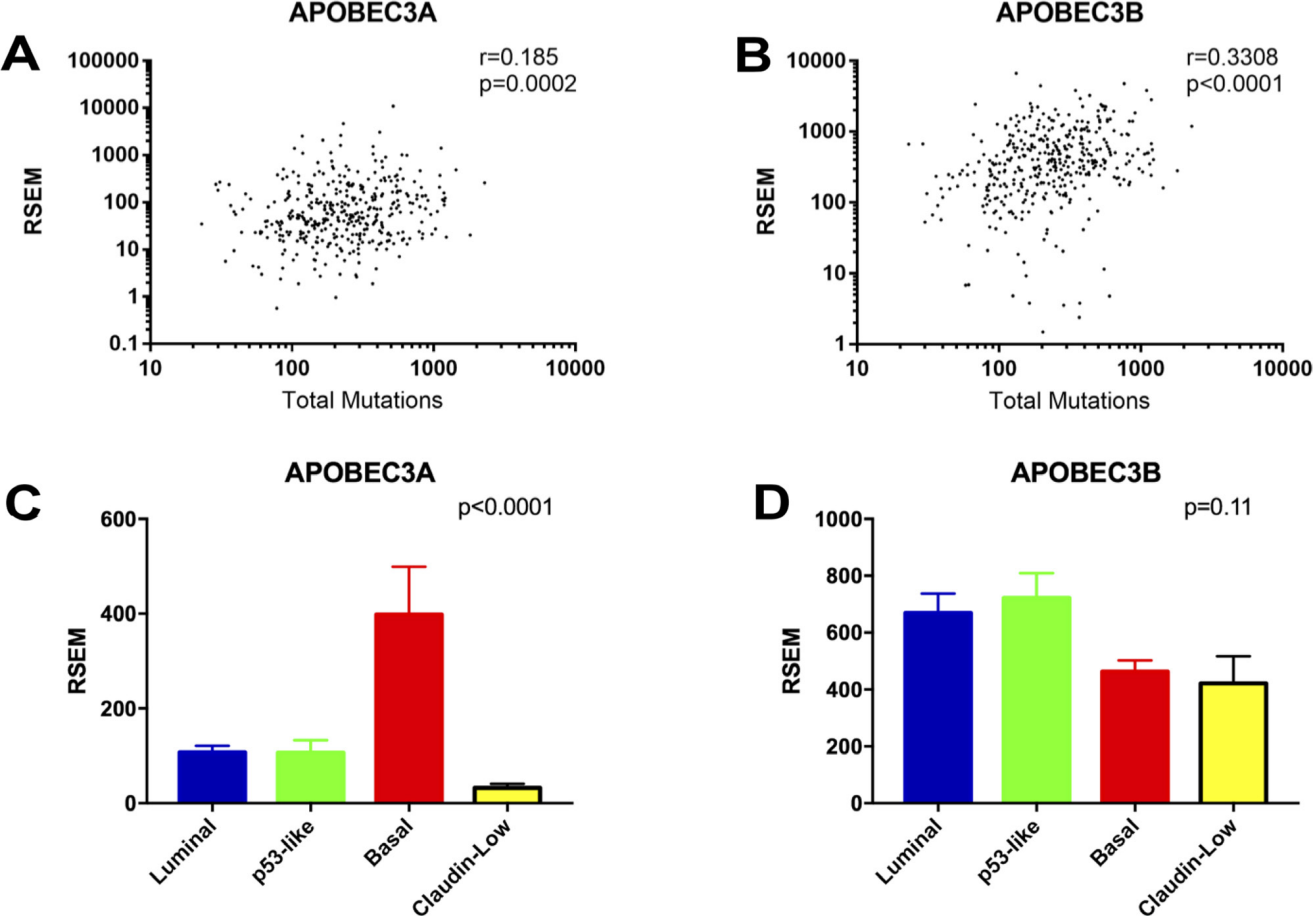
Correlations of *APOBEC3A* and *APOBEC3B* expression with mutational burden and molecular subtype (**A**) Spearman correlation between total mutations and *APOBEC3A* expression. (**B**) Spearman correlation between total mutations and *APOBEC3B* expression. (**C**) Expression of *APOBEC3A* in the molecular subtypes of bladder cancer. (**D**) Expression of *APOBEC3B* in the molecular subtypes of bladder cancer.

To determine the relationship between APOBEC mutagenesis, APOBEC gene expression, and molecular subtype, we next investigated the association of APOBEC3 enzyme expression with total mutations in both the entire TCGA bladder cancer dataset and in the four molecular subtypes (luminal, p53-like, basal, and claudin-low; Supplementary Figure 1). Expression of *APOBEC3A* and *APOBEC3B* were the only APOBEC enzymes that directly correlate with the total mutation burden in bladder cancer (Figure 4A–4B; Supplementary Figure 3). We found no association between total mutations or APOBEC enrichment score and the molecular subtypes of bladder cancer (Supplementary Figure 4). However, *APOBEC3A* is expressed at a significantly higher level in the basal subtype than in luminal, p53-like, or claudin-low subtypes (Figure 4C), while *APOBEC3B* is evenly expressed across subtypes (Figure 4D). *APOBEC3A* and *APOBEC3B* expression levels correlate with total mutations in every subtype (Supplementary Figure 5).

### Gene expression associated with APOBEC enrichment

**Figure 5:**
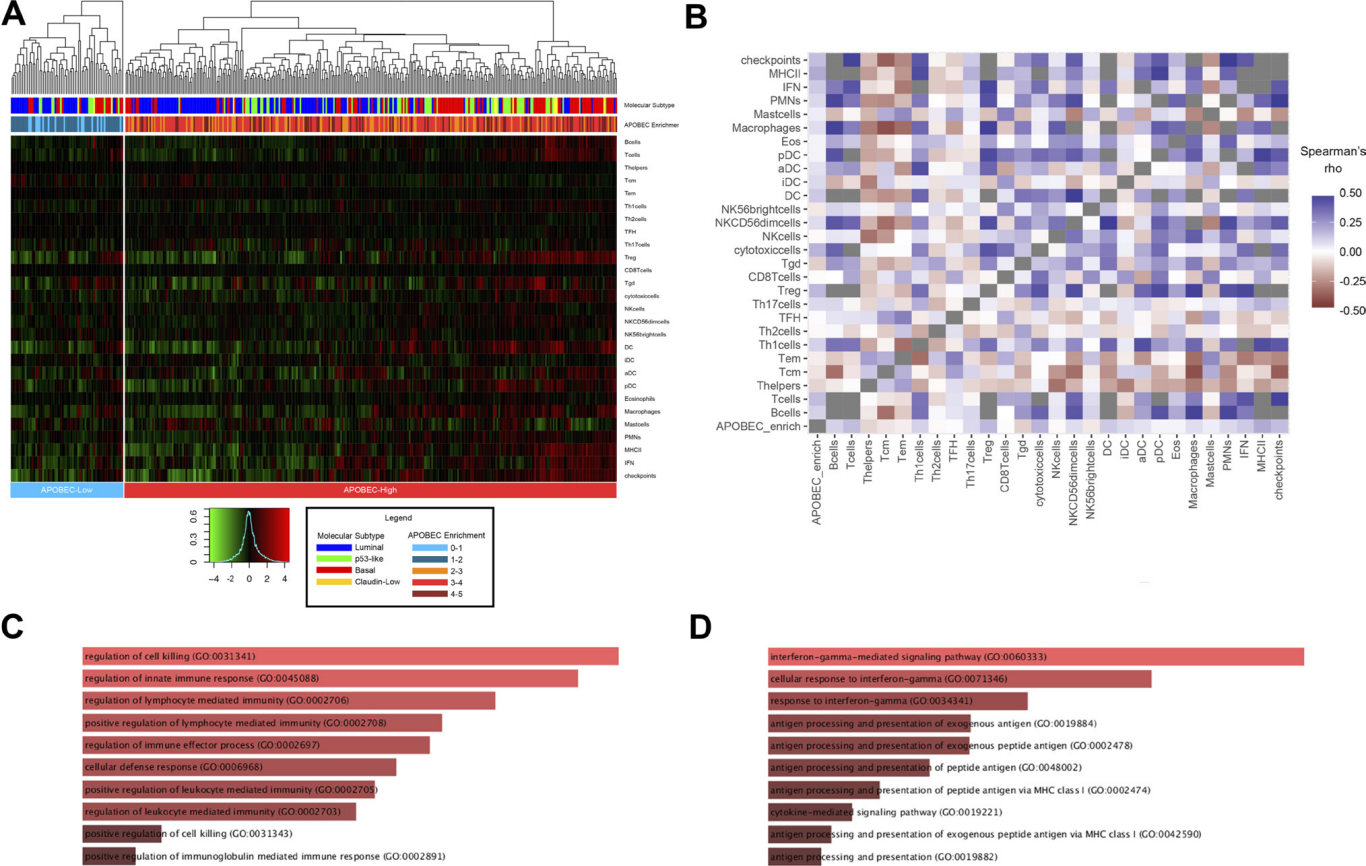
Association of APOBEC enrichment with immune signatures and gene expression (**A**) Heirarchical clustering of immune cell and checkpoint signatures in APOBEC-high and APOBEC-low tumors. Annotation of each tumor with molecular subtype and numeric APOBEC enrichment score is provided in the legend. (**B**) Correlation matrix of APOBEC enrichment score and immune signature. (**C**) Barplot of gene ontology biological processes for genes highly expressed in APOBEC-high tumors compared to APOBEC-low tumors. (**D**) Barplot of gene ontology biological processes for genes positively correlated with numeric APOBEC enrichment score. Barplots generated with Enrichr and bar size based on combined score of *p*-value and deviation from expected rank. [49, 50]. Tcm, central memory T cell; Tem, effector memory T cell; Th1, type 1 T helper cell; Th2, type 2 T helper cell; TFH, T follicular helper cell; Th17, T-helper 17 cell; Treg, regulatory T cell; Tgd, γδ T cell; NK, natural killer; DC, dendritic cell; iDC, immature dendritic cell; aDC, activated dendritic cell; pDC, plasmacytoid dendritic cell; PMNs, polymorphonuclear leukocytes, IFN, interferon; MHC II, major histocompatibility complex class II.

To investigate the potential functional mechanisms associated with APOBEC mutagenesis, we approached gene expression association with APOBEC enrichment in the TCGA cohort by several methods. First, we examined the association of APOBEC enrichment with 27 immune cell and immune marker gene expression signatures [21, 22]. APOBEC-high tumors demonstrate relative higher expression of these immune signatures, with basal and claudin-low tumors clustering near high expression of immune signatures (Figure 5A; differences in immune signatures between molecular subtypes Supplementary Figure 6). APOBEC enrichment score significantly correlates with B-cell, T-cell, Th1 T-cell, T-regulatory cell, γδ T-cell, cytotoxic T-cell, dendritic cell, MHC-II, IFN, and immune checkpoint signatures (Figure 5B; Supplementary Figure 7). This pattern is consistent across the molecular subtypes of bladder cancer (Supplementary Figure 8).

We next analyzed differentially expressed genes between the 324 APOBEC-high and 64 APOBEC-low tumors. APOBEC-high tumors were enriched for expression of genes related to regulation of the immune response and lymphocyte-mediated immunity (Figure 5C; Supplementary Table 5), whereas APOBEC-low tumors demonstrated higher expression of genes related to transcription and translation (Supplementary Table 6). Similarly, correlation between continuous numeric APOBEC enrichment score and gene expression revealed a positive relationship between APOBEC enrichment score and gene families involved in IFNγ signaling, antigen presentation, and regulation of the immune response, including the immune checkpoint *HAVCR2* (also known as TIM-3; Spearman *r* = 0.229, *p <* 0.0001) (Figure 5D; Supplementary Table 7), while genes inversely correlated with APOBEC enrichment score were enriched in processes related to transcription and translation (Supplementary Table 8).

Finally, to further evaluate the association of APOBEC mutational pattern, *APOBEC3B* enzyme expression, and the immune environment, we analyzed *APOBEC3B* expression in two APOBEC-low cell lines (RT4 and KU-19-19) and two APOBEC-high cell lines (HT-1376 and UM-UC-3) after exposure to IFNγ. Expression of *APOBEC3B* increased after exposure to IFNγ in APOBEC-high cell lines, but not in APOBEC-low cell lines suggesting that urothelial cancers with high APOBEC activity may have a feed-forward mechanism resulting in increased APOBEC expression upon immune activation (*p* = 0.03, Supplementary Figure 9).

## DISCUSSION

APOBEC mutagenesis is the predominant mutational pattern in bladder cancer. In this paper, we demonstrate that tumors enriched for APOBEC mutagenesis (APOBEC-high tumors) have better survival and are more likely to have mutations in DNA damage repair genes and chromatin regulation genes, while tumors not featuring the APOBEC mutational pattern (APOBEC-low tumors) are significantly more likely to harbor mutations in *FGFR3* and *KRAS/HRAS/NRAS*, which are mutually exclusive. Expression of *APOBEC3A* and *APOBEC3B* correlates with overall mutation load in bladder cancer, regardless of molecular subtype. In addition, APOBEC enrichment is associated with immune signatures and upregulation of immune-related genes including interferon signaling.

Our work is consistent with several prior studies linking APOBEC expression to mutational burden, survival, and DNA damage. *APOBEC3B* expression is upregulated in breast cancer and is associated with total mutation burden [5]. Overexpression of *APOBEC3B* in breast cancer cell lines results in DNA fragmentation, increased C>T mutations, delayed cell cycle arrest, and eventual cell death [5]. Furthermore, knockdown of *APOBEC3B* with short hairpin RNA in breast cancer cell lines decreases total number of uracil lesions, *TP53* mutations, and C>T mutations [5]. In HEK-293 cell lines with inactivated *TP53*, overexpression of *APOBEC3B* induces markers of DNA damage response and leads to a kataegic mutational pattern in the APOBEC-targeting TCW motif [15].

*APOBEC3A* expression was not initially detectable in breast cancer cell lines [5], and *APOBEC3B* expression correlates strongly with overall mutations in multiple malignancies [5, 14], leading many to initially believe that APOBEC3B is responsible for the majority of the APOBEC mutational signature. However, *APOBEC3A* expression is also correlated with mutational burden [3], as we demonstrate again here, *APOBEC3A* is highly proficient at cytidine hypermutation and creation of DNA double-strand breaks [23, 24], and may have a larger role in mutagenesis than previously recognized [11].

Middlebrooks *et al*. demonstrated that expression of both *APOBEC3A* and *APOBEC3B* can be induced in bladder cancer cell lines by bleomycin, a DNA damaging agent, and by an RNA virus that induces an interferon response [4]. This group also used the TCGA bladder cancer dataset to demonstrate that APOBEC mutagenesis is associated with improved overall survival, and that APOBEC-high tumors are enriched for *TP53* and *PIK3CA* mutations [4]. We demonstrate that bladder tumors not enriched for APOBEC mutagenesis frequently harbor mutations in *FGFR3* or the RAS family of oncogenes, and we also demonstrate that *APOBEC3A* is expressed at significantly higher levels in the basal subtype of bladder cancer, compared to other subtypes.

The proposed substrate for APOBEC mutagenesis is ssDNA, a common DNA repair intermediate that may accumulate in cells with defects in DNA repair pathways [25]. APOBEC-high tumors are more likely to have mutations in genes related to DNA repair and chromatin regulation, including *TP53*, *NCOR1*, *MLL3* (*KMT2C*), *MLL* (*KMT2A*), *ATR*, *BRCA2*, and *ARID1A* in the TCGA dataset. In the smaller BGI dataset, APOBEC-high tumors are more likely to have mutations in *TP53* and *ARID1A*. We also demonstrate a higher frequency of *PIK3CA* mutations in APOBEC-high tumors in both the TCGA and BGI datasets. *PIK3CA* has been previously reported to be mutated at a high frequency in specific TCW-containing helical motifs across a number of tumor types [4, 26]. Our analysis supports these results, with the majority of *PIK3CA* mutations in APOBEC-high tumors occurring in the helical domain at E542 and E545. These specific mutations in E542 and E545 have also been reported as hotspot mutations in breast cancer [27]. The location of these mutations in APOBEC motifs suggests that these *PIK3CA* mutations may be a result of APOBEC-mediated mutagenesis, rather than supporting or driving APOBEC-mutagenesis.

Interestingly, APOBEC-low tumors in this study were more likely to be low-grade and have mutations in *FGFR3* and the RAS family of oncogenes. This data was confirmed in the mutational analysis of 140 non-muscle-invasive bladder cancers [28]. This suggests that tumors not enriched for the APOBEC mutational pattern may be driven by oncogenes which may dysregulate cellular homeostasis via mechanisms that do not result in accumulation of ssDNA intermediates used as substrate for APOBEC mutagenesis. Alternatively, APOBEC-mediated mutagenesis may arrest tumor cells that do not possess inactivated *TP53* or other tumor suppressors [15].

We also found a lower level of APOBEC-mediated mutagenesis in patients of Asian ethnicity in the TCGA cohort, compared to those of non-Asian ethnicity. In addition, a lower percentage of tumors from the BGI cohort were enriched for APOBEC-mediated mutagenesis compared to the TCGA cohort. One explanation is that patients of Asian ethnicity in the TCGA cohort express lower levels of *APOBEC3B.* Alternatively, non-APOBEC genetic instability processes may be present in these patients. In the BGI cohort, COSMIC Signature 22, which is a non-APOBEC mutational signature, was observed in several patients in both the APOBEC-high and APOBEC-low groups. This signature is attributed to aristolochic acid, which is found in plants of the *Aristolochia* genus and many of which are used in traditional Chinese herbal medicine [29].

**Figure 6:**
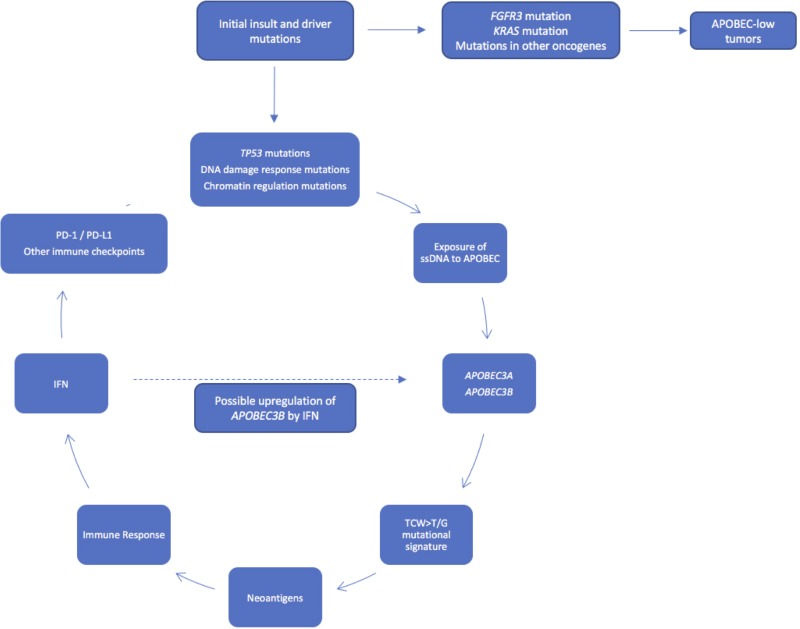
Working model of APOBEC-mediated mutagenesis in bladder cancer Accumulation of mutations in *TP53, ATR*, *BRCA2*, and/or other DNA damage response genes or chromatin regulation genes may expose more substrate ssDNA to *APOBEC3A* and *APOBEC3B*, leading to a high level of APOBEC-mediated mutagenesis and a hypermutation phenotype, with subsequent neoantigen burden, immune response, and survival benefit. Tumors with mutations in *FGFR3* and *KRAS* may not expose enough substrate to APOBEC enzymes to promote APOBEC-mediated mutagenesis.

Based on our results and the above discussion, we propose a working model of mutagenesis and the immune response in bladder cancer (Figure 6), in which a urothelial cell acquires one or more driver mutation(s). Accumulation of mutations in *TP53*, *ARID1A*, *ATR*, *BRCA2,* and/or other DNA damage response genes or chromatin regulation genes may result in the accumulation of ssDNA substrate for *APOBEC3A* and *APOBEC3B*, leading to a high level of APOBEC-mediated mutagenesis and a hypermutation phenotype. This hypermutation in turn leads to a large neoantigen burden and the subsequent immune response generated from this increase in neoantigens. In addition, APOBEC enzymes may be induced and overexpressed in response to interferon [4, 9, 24, 30], potentially causing a positive feedback loop in tumor cells enriched for APOBEC mutagenesis. In contrast, other tumors with mutations in the *FGFR3*/RAS pathway or other oncogenes may not expose sufficient substrate ssDNA to APOBEC enzymes to undergo significant APOBEC mutagenesis. These APOBEC-low tumors have poor survival, despite an enrichment for *FGFR3* mutations and low-grade tumors, which were classically considered more benign phenotypes.

Several limitations of this study warrant mention. We utilized multiple datasets for analysis, but the only dataset with both whole genome sequencing and RNA expression data is TCGA; therefore correlative data between mutational patterns and gene expression, including analysis in molecular subtypes and immune signatures, warrants replication. In addition, TCGA does not currently include any systemic treatment-related information. However, mutations in *ERCC2* [31, 32] and other DNA repair genes [33, 34] are associated with response to platinum-based therapy, and further investigation into the role of APOBEC mutagenesis and response to both cytotoxic chemotherapy and immunotherapy is warranted. In addition, while we demonstrate that treatment of APOBEC-high cell lines with IFNγ leads to upregulation of *APOBEC3B*, inherent differences in morphology or other non-measured genes could also explain differences. It is also possible that other pro-inflammatory cytokines may have the same effect; this is currently under investigation. Another limitation is the lack of a specific gene expression signature observed in APOBEC-low tumors other than transcription- and translation-related genes, potentially due to the heterogeneity of this group. In addition, gene expression correlations with APOBEC enrichment score in APOBEC-low tumors would not be expected to generate a strong signal, as these tumors by definition have a low and heterogeneous numerical APOBEC enrichment score.

In summary, APOBEC enzymes are a major source of mutation in bladder cancer. Tumors enriched for APOBEC mutagenesis have better survival and are more likely to have mutations in DNA damage repair genes and chromatin modifying genes. The APOBEC mutagenesis signature is associated with immune signatures and with increased expression of immune-related genes. Bladder tumors not enriched for APOBEC mutagenesis are more likely to have mutations in *FGFR3* and the RAS family of oncogenes, which are mutually exclusive, and these patients have poor overall survival. Further study of the regulation of APOBEC enzymes, mutagenesis, and response to subsequent therapy may provide further insight into the mutational landscape and potential therapeutics for bladder cancer.

## METHODS

### The Cancer Genome Atlas

The Cancer Genome Atlas (TCGA) bladder urothelial carcinoma data was downloaded from the Broad Institute Genome Data Analysis Center (GDAC) (http://gdac.broadinstitute.org) [20, 35]. Data from GDAC was downloaded on November 8, 2016, from the analysis timestamp “analyses_2016_01_28” (doi:10.7908/C19G5M58) [20]. Downloaded data includes clinical and demographic data (age, sex, tumor stage, overall survival), mutation annotation files (MutSig 2CV v3.1; MAF file; Mutsig_maf_modified.maf.txt) and mRNA expression (Illumina HiSeq RNAseqV2). TCGA RNA-seq mRNA expression levels are presented as RNA-seq by expectation-maximization (RSEM) values [36].

Clinical information was available on 412 TCGA bladder cancer samples, RNA-seq data was available on 408 samples, and mutation information was available on 395 samples. Overlap between the 412 patients with clinical information, 408 patients with RNA-seq data, and 395 patients with mutation annotation information yields 391 patients. Three outliers were removed from mutational analysis (TCGA-DK-A6AW, >150 mutations/Mb; TCGA-XF-AAN8 and TCGA-FD-A43, both with ≤5 total mutations).

### Beijing Genomics Institute and Cancer Cell Line Encyclopedia

Mutational and staging data from the BGI (*n* = 99 samples) [37] was downloaded from cBioPortal (http://cbioportal.org) [38]. No survival or expression data is available for the BGI dataset. Mutational data from the CCLE was downloaded from the Broad CCLE portal (http://www.broadinstitute.org/ccle) [39].

### Mutation analysis and APOBEC enrichment

Analysis and visualization of mutations was performed using R v3.3.3, Bioconductor [40] v3.4 (http://www.Bioconductor.org), and MAFtools v1.0.55 [41]. Mutation rates per sample were calculated using MutSig2CV v3.1 from the Broad Institute GDAC [1]. APOBEC enrichment score based on the frequency of TCW>T/G mutations was calculated as previously described [3, 4, 19]. Samples were classified into two groups: “APOBEC-high” based on APOBEC enrichment > 2 and Benjamini-Hochberg false-discovery-rate corrected *p*-value < 0.05; and “APOBEC-low” based on an APOBEC enrichment < 2 and/or Benjamini-Hochberg false-discovery-rate corrected *p*-value ≥ 0.05. Survival outcomes between patients with APOBEC-high-enrichment and APOBEC-low-enrichment was performed using log-rank test and Kaplan-Meyer curves (R packages survival v2.41-2, survminer v0.3.1, ggplot2 v2.2.1). Significantly differentially mutated genes between APOBEC-high-enrichment and APOBEC-low-enrichment groups was performed using MAFtools [41] as previously described [42] and visualized with oncoplots and forest plots.

### Molecular subtyping

Molecular subtyping of 408 TCGA bladder urothelial carcinoma samples with RNA-seq data was performed using multiClust v1.4.0 [43]. Samples were classified as luminal, p-53-like, basal, or claudin-low as previously described [44] with hierarchical clustering using Euclidean distance and Ward's linkage method (ward.D2; heatmap shown in Supplementary Figure 1). Differences in mutational load and expression of APOBEC3 enzymes (RSEM) between tumor subtypes was compared using ANOVA.

### Immune signatures and gene expression associated with APOBEC enrichment

Immune signatures were calculated as previously described [22] using previously 24 published immune cell gene signatures from Bindea *et al*., [21] additional MHC-II and IFN gene signatures from Faruki *et al*., [22] and a immune checkpoint gene signature consisting of PD-1, PD-L1, PD-L2, CTLA-4, B7-H4, TIM-3, LAG3, OX40, and OX40L. Hierarchical clustering of immune signatures in APOBEC-high and APOBEC-low tumors was performed with multiClust v1.4.0 [43]. Differential expression analysis between TCGA APOBEC-high and APOBEC-low tumors was performed using limma v3.30.13 and edgeR v3.16.5 [45, 46]. Association of immune signatures and gene expression with numeric APOBEC enrichment score was performed using Spearman's rho. Differences in immune signatures between tumor subtypes was compared using ANOVA. Functional annotation of genes was performed with DAVID v6.8 (http://david.ncifcrf.gov) [47, 48] and visualized with Enrichr (http://amp.pharm.mssm.edu/Enrichr/) [49, 50].

### Cell lines and qPCR

Four bladder cancer cell lines (RT-4, KU-19-19, HT-1376, and UM-UC-3) were cultured in standard media (McCoy’s, RPMI1640, EMEM, and EMEM, respectively) with 10% FBS and 1% penicillin/streptomycin (Thermo Fisher Scientific) with the addition of either IFNγ (10 ng/mL; R&D Systems) or the equivalent amount of sterile PBS for 48 h. RT-4, HT-1376, and UM-UC-3 were obtained from ATCC, KU-19-19 was obtained from DSMZ. All experiments were performed on passages 4–10. Cells were lysed with TRIzol (Thermo Fisher Scientific) and total RNA was isolated following standard Abcam RNA isolation protocol. Quality and quantity of isolated RNA was evaluated with NanoDrop 2000 (Thermo Fisher Scientific). cDNA was prepared using standard amounts of RNA per sample (600 ng; 250 ng/uL) with the TaqMan RT Reagents kit (Thermo Fisher Scientific). Samples were run in technical triplicate in a 20-μL SsoAdvanced Universal Probes Supermix (BioRad) in standard 96-well PCR plates. Expression level of *APOBEC3B* and endogenous control *GAPDH* was measured using BioRad PrimePCR Probe Assays (qHsaCIP0039581 and qHsaCEP0041396) using BioRad CFX Connect RT-PCR Detection System. Experiment was repeated in triplicate before analysis, and the ΔΔCt method was used to calculate differences in gene expression between IFNγ-stimulated cells and controls.

## SUPPLEMENTARY MATERIALS FIGURES AND TABLES







## Abbreviations and acronyms

TCGA: The Cancer Genome Atlas
BGI: Beijing Genomics Institute
CCLE: Cancer Cell Line Encyclopedia
COSMIC: catalogue of somatic mutations in cancer
ssDNA: single stranded DNA
APOBEC: apolipoprotein B mRNA editing catalytic polypeptide-like
GCAC: Genome Data Analysis Center
MAF: mutation annotation format
“APOBEC-high”: tumors enriched for APOBEC mutagenesis
“APOBEC-low”: tumors not enriched for APOBEC mutagenesis.
