# Supplementary material for: APOBEC-mediated mutagenesis in urothelial carcinoma is associated with improved survival, mutations in DNA damage response genes, and immune response
Source: Oncotarget. 2017 Dec 16;9(4):4537–48. doi: 10.18632/oncotarget.23344 (PMC5796993; doi:10.18632/oncotarget.23344)
Supplement: Supplementary file 1 [file oncotarget-09-4537-s001.pdf]

APOBEC-mediated mutagenesis in urothelial carcinoma is associated with improved survival, mutations in DNA damage response genes, and immune response

SUPPLEMENTARY MATERIALS

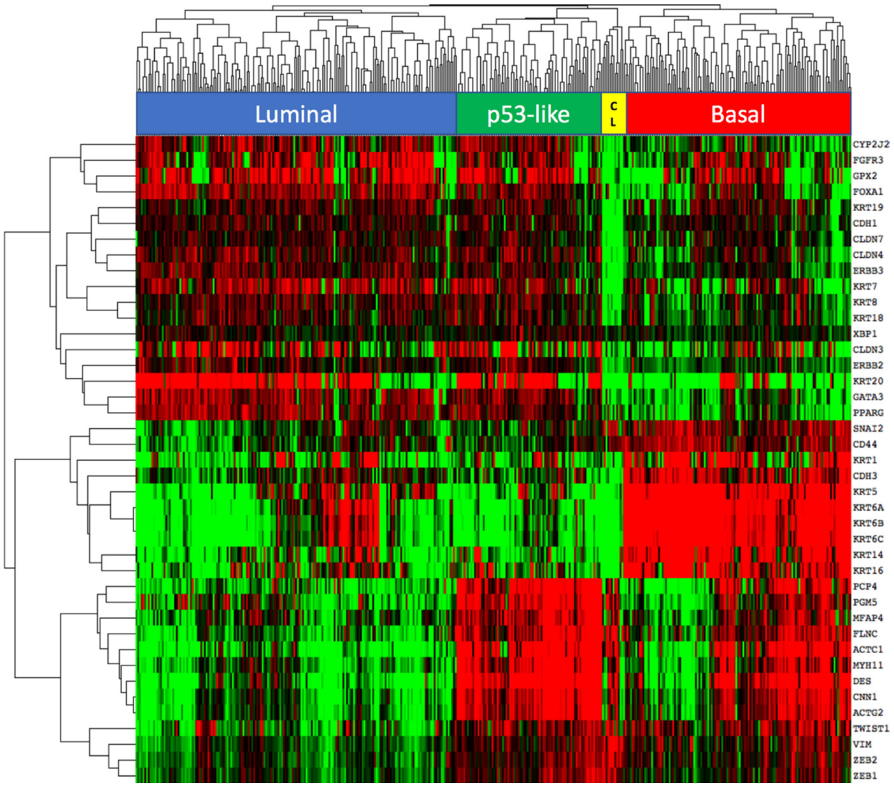

Supplementary Figure 1: Heirarchical clustering of TCGA bladder cancer samples into luminal, p53-like, claudin-low (CL), and basal subtypes, with heatmap displaying relative expression of gene sets used to characterize each subtype. [31].

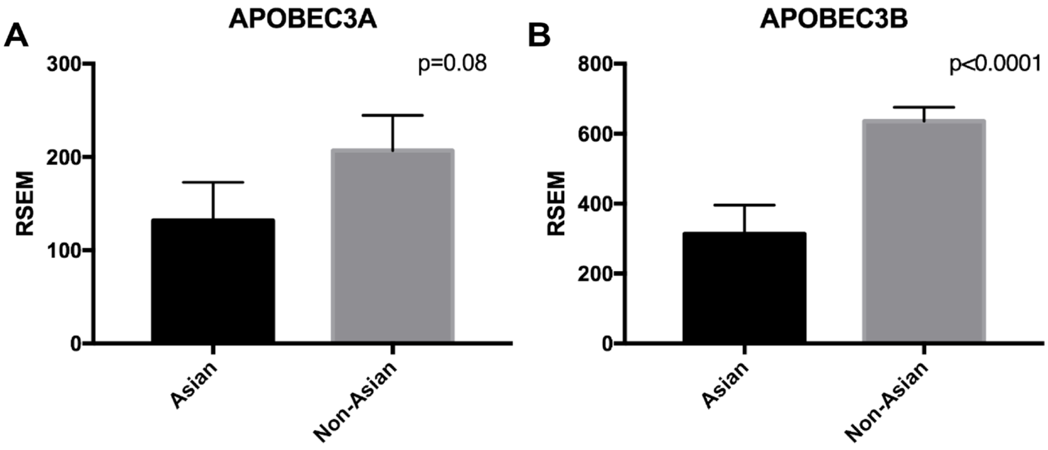

Supplementary Figure 2: APOBEC3 expression in TCGA bladder cancer patients based on ethnicity, grouped into Asian patients and non-Asian patients. (**A**) *APBOEC3A* expression (**B**) *APOBEC3B* expression.

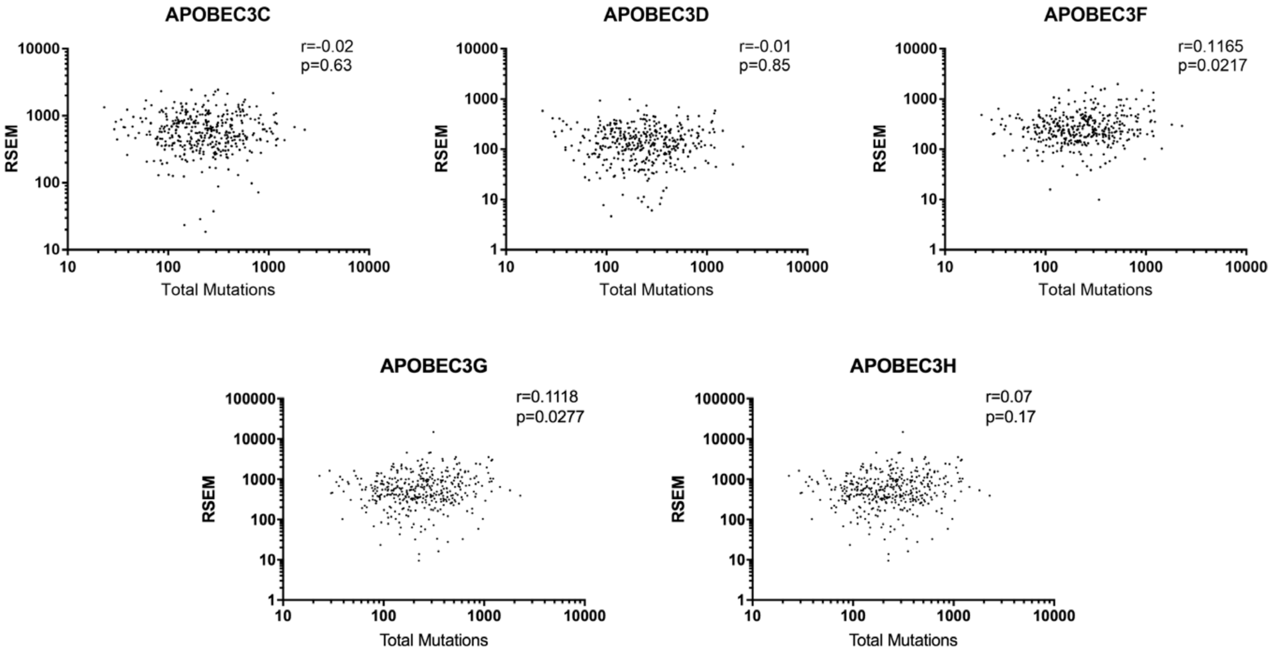

Supplementary Figure 3: Correlation of *APOBEC3C, APOBEC3D, APOBEC3F, APOBEC3G*, and *APOBEC3H* mRNA expression with total mutations in bladder cancer.

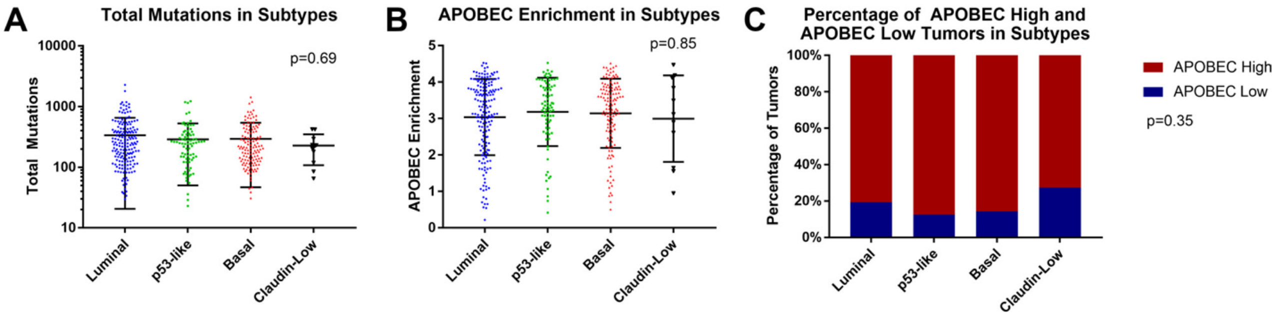

Supplementary Figure 4: (**A**) The number of total mutations does not differ among molecular subtypes of bladder cancer. (**B**) APOBEC enrichment score does not differ among molecular subtypes of bladder cancer. (**C**) The proportion of APOBEC-high and APOBEC-low tumors does not differ among molecular subtypes of bladder cancer.

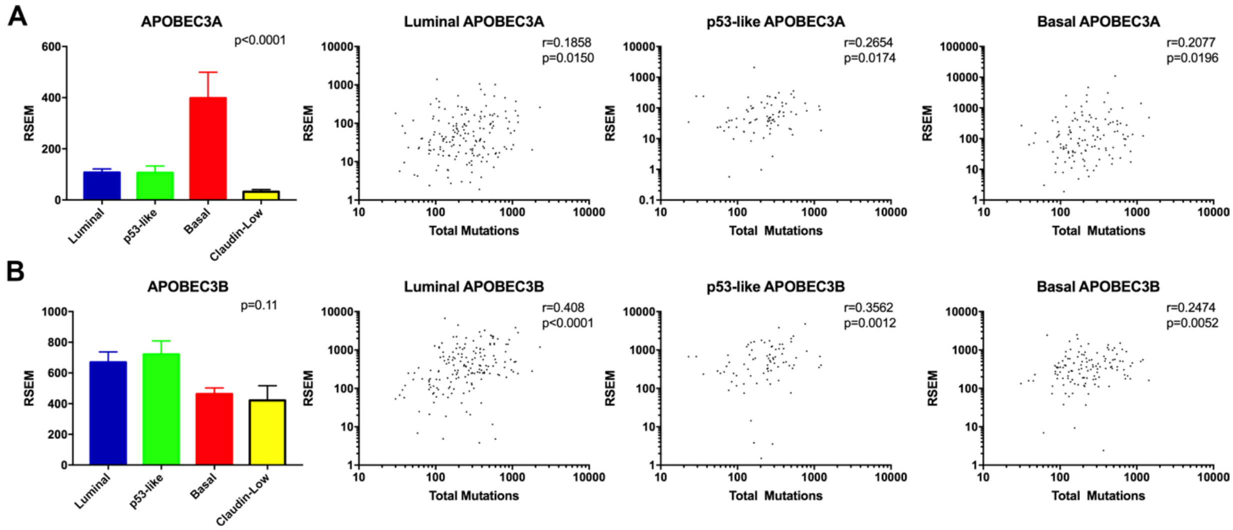

Supplementary Figure 5: **(A)** *APOBEC3A* is expressed at significantly higher levels in the basal subtype of bladder cancer, but expression correlates with total mutations in luminal, p53-like, and basal subtypes (**B**) *APOBEC3B* is expressed at significantly higher levels in the basal subtype of bladder cancer, but expression correlates with total mutations in luminal, p53-like, and basal subtypes. (Claudin-low subtype excluded from correlations due to small number of patients).

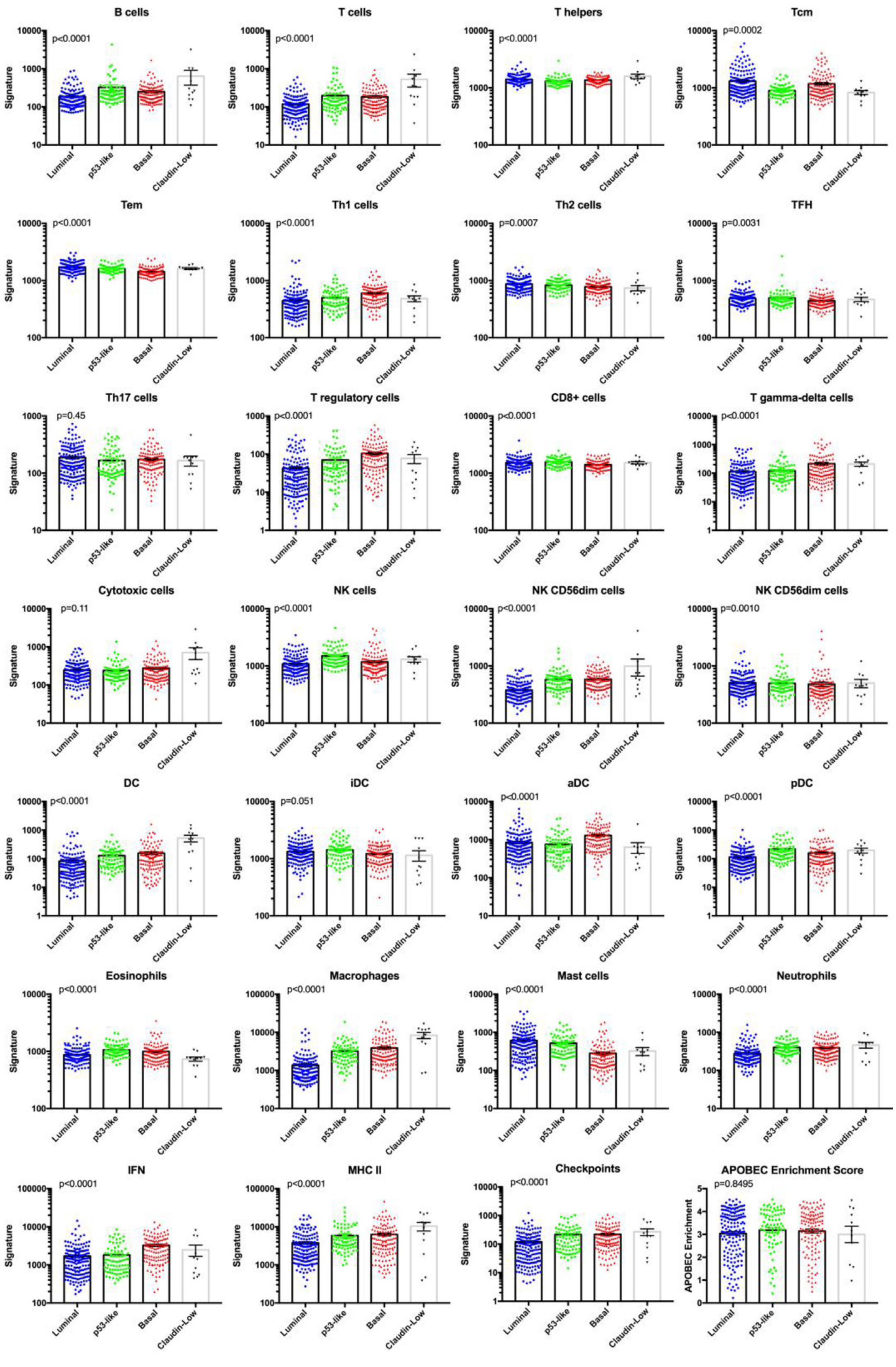

Supplementary Figure 6: Immune signatures in the molecular subtypes of bladder cancer. Each immune signature is demonstrated along the Y axis, with the molecular subtypes of bladder cancer on the X axis. Differences in signature between subtypes compared using ANOVA. For illustration, APOBEC enrichment score between subtypes is shown as the last figure.

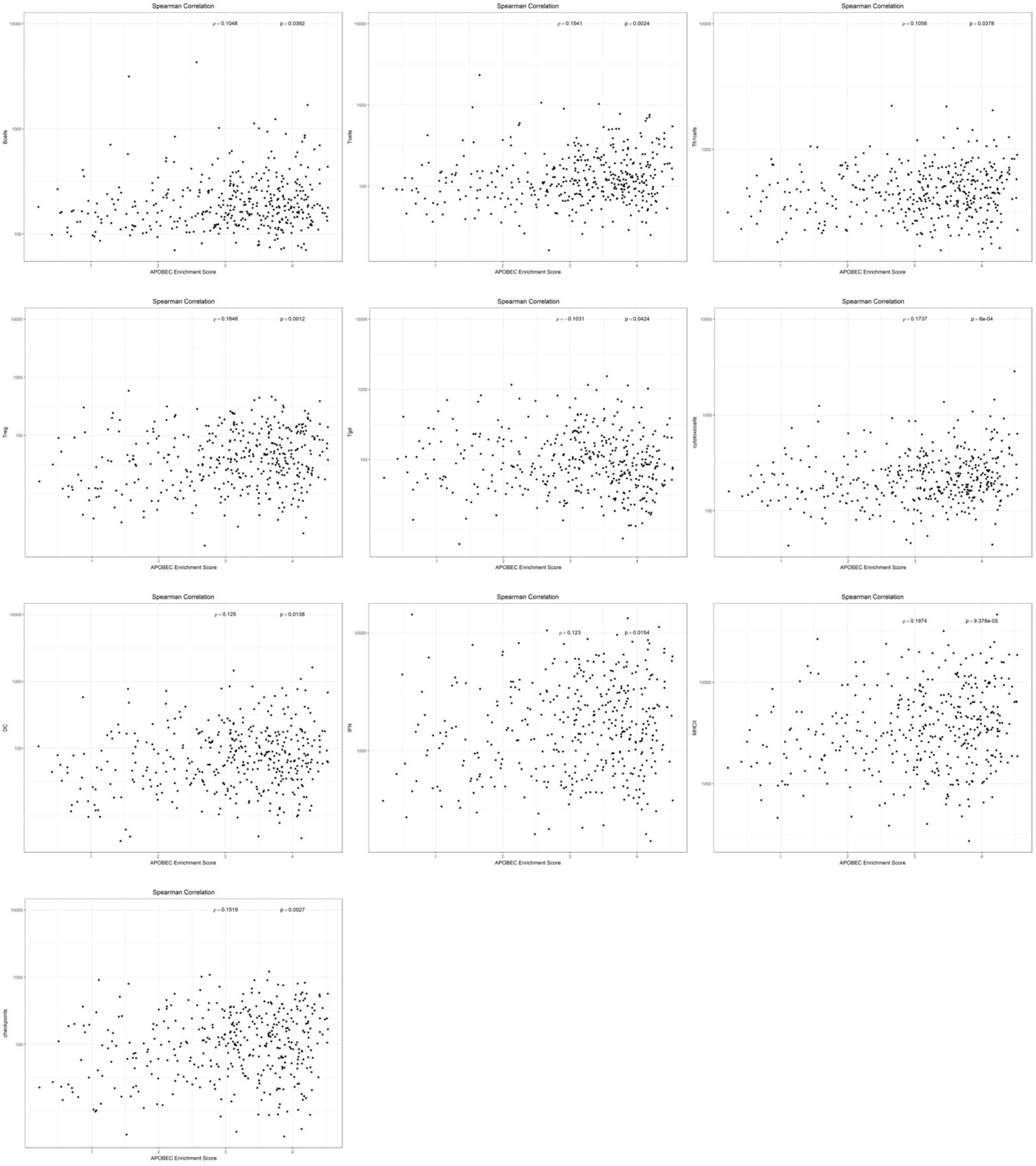

Supplementary Figure 7: APOBEC enrichment correlates with immune signatures. X-axis: APOBEC enrichment score, Y-axis, immune signature. Th1, type 1 T helper cell; Treg, regulatory T cell; Tgd, γδ T cell; DC, dendritic cell; IFN, interferon; MHC II, major histocompatibility complex class II.

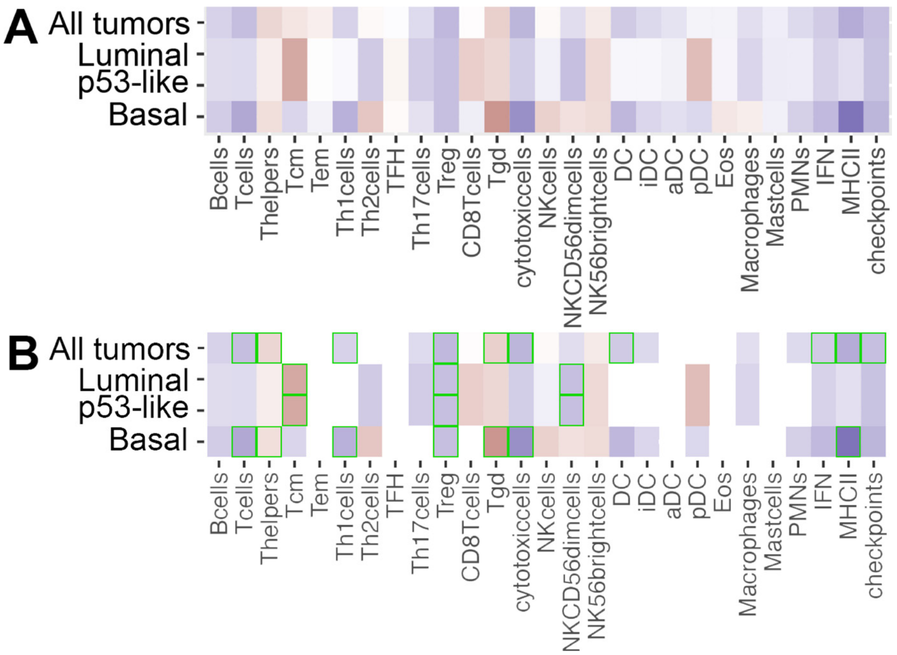

Supplementary Figure 8: Correlation matrix of APOBEC enrichment score and immune signature across subtypes. (**A**) Association between APOBEC enrichment score in all tumors, luminal tumors, p53-like tumors, and basal tumors compared against immune signatures using Spearman's rho. (Claudin-low subtype excluded from correlations due to small number of patients). (**B**) Significant correlations outlined in green boxes. Luminal Tcm (rho = –0.1934, p = 0.0114), Treg (rho = 0.1516, p = 0.0478), NKCD56dim (rho = 0.1507, p = 0.0492), checkpoints (rho = 0.1419, p = 0.064); p53-like Tcm (rho = –0.1934, p = 0.01135), Treg (rho = 0.1516, p = 0.0478), NKCD56dim (rho = 0.1507, p = 0.0492); Basal Bcells (rho = 0.1048, p = 0.0392), Tcells (rho = 0.1541, p = 0.0024), Th1cells (rho = 0.1056, p = 0.0378), Treg (rho = 0.1646, p = 0.0012), Tgd (rho = –0.1031, p = 0.0424), Cytotoxic (rho = 0.1737, p = 0.0006), MHCII (rho = 0.3391, p = 0.0001). Tcm, central memory T cell; Tem, effector memory T cell; Th1, type 1 T helper cell; Th2, type 2 T helper cell; TFH, T follicular helper cell; Th17, T-helper 17 cell; Treg, regulatory T cell; Tgd, γδ T cell; NK, natural killer; DC, dendritic cell; iDC, immature dendritic cell; aDC, activated dendritic cell; pDC, plasmacytoid dendritic cell; PMNs, polymorphonuclear leukocytes, IFN, interferon; MHC II, major histocompatibility complex class II..

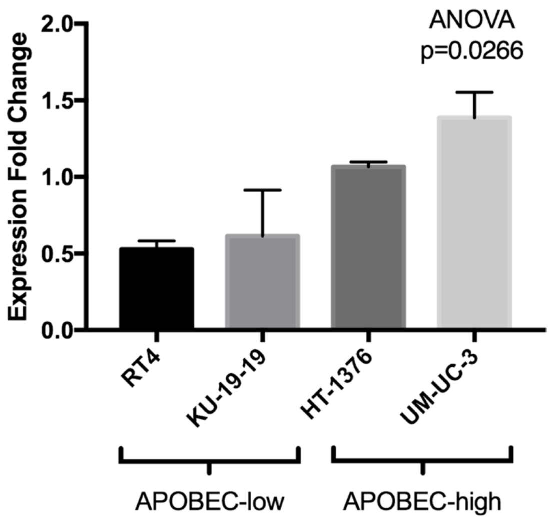

Supplementary Figure 9: Relative *APOBEC3B* expression following exposure to IFNγ (10 ng/mL). Columns represent mean ± SEM for three qPCR reactions. Ct values were standardized against the housekeeping gene GAPDH, and fold change was calculated as $2^{(-DDCt)}$, relative to a control cell line not stimulated with IFNγ.

Supplementary Table 1A: Clinical variables in TCGA APOBEC-high and APOBEC-low tumors

Variable	APOBEC High (*n* = 324)	APOBEC Low (*n* = 64)	*P*-value
Age, years	68.4 ± 10.4	66.4 ± 11.5	0.17
Gender			
Male	242 (75%)	46 (72%)	0.80
Female	81 (25%)	18 (28%)	
NA	1 (0%)	0	
Race/Ethnicity			
White	273 (84%)	40 (62%)	**<0.0001**
Black/African American	16 (5%)	6 (9%)	
Asian	22 (7%)	17 (26%)	
NA	13 (4%)	4 (6%)	
Cluster			
Basal	108 (33%)	18 (28%)	0.351
Claudin Low	8 (2%)	3 (5%)	
Luminal	138 (42%)	33 (52%)	
p53-like	70 (22%)	10 (16%)	
T stage			
NA	28 (9%)	5 (8%)	0.073*
T0	0	1 (2%)	
T1	2 (1%)	1 (2%)	
T2	86 (26%)	24 (38%)	
T3	164 (51%)	21 (33%)	
T4	44 (14%)	12 (19%)	
N-stage			0.48
N0	177 (55%)	43 (67%)	
N1	38 (12%)	6 (9%)	
N2	65 (20%)	9 (14%)	
N3	7 (2%)	1 (2%)	
NA	37 (11%)	5 (8%)	
Stage			
NA	2 (1%)	1 (2%)	0.38
Stage I	1 (0%)	1 (2%)	
Stage II	96 (30%)	24 (38%)	
Stage III	112 (35%)	20 (31%)	
Stage IV	113 (35%)	18 (28%)	
Grade			
High grade	311 (96%)	53 (83%)	**<0.0001**
Low grade	9 (3%)	11 (17%)	
NA	4 (1%)	0	
Smoking Category			0.53
NA	11	2	
1	86	17	
2	65	19	
3	90	17	
4	61	8	
5	11	1	

*T2, T3, T4 only

Supplementary Table 1B: Demographics and clinical characteristics in the Beijing Genomics Institute dataset

**Variable**	**BGI APOBEC High (*n* = 69)**	**BGI APOBEC Low (*n* = 30)**	***P*-value**
Age, years	64.2 ± 12.2	57.7 ± 14.3	**0.022**
Gender			
Male	62 (90%)	26 (87%)	0.73
Female	7 (10%)	4 (13%)	
Grade			0.43
Grade 1	26 (38%)	15 (50%)	
Grade 2	27 (39%)	8 (27%)	
Grade 3	16 (23%)	7 (23%)	
Stage			
Superficial (Ta, T1)	22 (32%)	15 (50%)	0.12
Invasive (T2, T3, T4)	47 (68%)	15 (50%)	
T-Stage			
Ta	5 (7%)	1 (3%)	0.15
T1	17 (25%)	15 (50%)	
T2	22 (32%)	6 (20%)	
T3	15 (22%)	6	
T4	10 (14%)	2	
N-Stage			
N0	66 (96%)	28	0.64
N1	3 (4%)	2	

Supplementary Table 2: Significantly Mutated Genes between TCGA APOBEC-high and APOBEC-low groups. See Supplementary_Table_2

Supplementary Table 3: Gene ontology biological processes for genes frequently mutated in TCGA APOBEC-high bladder tumors

Term	*P* Value	Genes	Fold enrichment	Benjamini
GO:0016569~covalent chromatin modification	3.67E-05	CHD9, CHD7, BPTF, PBRM1, ARID1A, NCOR1	15.64229157	0.018751321
GO:0006338~chromatin remodeling	1.92E-04	CHD7, BPTF, INO80, PBRM1, ARID1A	17.12770298	0.048395942
GO:0060285~cilium-dependent cell motility	5.89E-04	DNAH17, DNAH7, DNAH8	80.34449761	0.096422305
GO:0006974~cellular response to DNA damage stimulus	0.005080679	UBR5, TP53, ATR, POLQ, APC	7.081646424	0.481636357
GO:0034644~cellular response to UV	0.009446977	TP53, INO80, ATR	20.0861244	0.62452458
GO:0021545~cranial nerve development	0.009972028	CHD7, ERBB3	196.3976608	0.577640753
GO:0006302~double-strand break repair	0.020444055	BRCA2, INO80, POLQ	13.3907496	0.78186409
GO:0006511~ubiquitin-dependent protein catabolic process	0.022816152	UBR5, UBE4B, RNF213, TTC3	6.474648159	0.774331964
GO:0000724~double-strand break repair via homologous recombination	0.025311771	BRCA2, INO80, POLQ	11.943101	0.770049321
GO:0007420~brain development	0.025499997	BPTF, PLXNB2, BRCA2, RELN	6.202031394	0.73627992
GO:0032886~regulation of microtubule-based process	0.029623975	MACF1, APC	65.46588694	0.756011142
GO:0090399~replicative senescence	0.039305764	TP53, ATR	49.0994152	0.821695231
GO:0035418~protein localization to synapse	0.042511895	BSN, RELN	45.32253711	0.821704406
GO:0006281~DNA repair	0.04361875	UBR5, INO80, ATR, POLQ	5.014408361	0.806751581
GO:0031175~neuron projection development	0.043917732	CAMSAP1, UBE4B, FRY	8.837894737	0.786677299
GO:0003222~ventricular trabecula myocardium morphogenesis	0.052067366	CHD7, UBE4B	36.8245614	0.821731933
GO:0006978~DNA damage response, signal transduction by p53 class mediator resulting in transcription of p21 class mediator	0.052067366	TP53, BRCA2	36.8245614	0.821731933
GO:0010628~positive regulation of gene expression	0.056886171	APOB, ERBB3, ANK3, TP53	4.497656355	0.830976917
GO:0006461~protein complex assembly	0.057228464	PARD3, TP53, APC	7.618874773	0.815363068
GO:0032465~regulation of cytokinesis	0.070898187	BRCA2, BIRC6	26.7814992	0.864272869
GO:0010165~response to X-ray	0.070898187	TP53, BRCA2	26.7814992	0.864272869
GO:0007569~cell aging	0.080175331	TP53, BRCA2	23.5677193	0.884231511
GO:0061098~positive regulation of protein tyrosine kinase activity	0.083247448	ERBB3, RELN	22.66126856	0.881837178
GO:0043001~Golgi to plasma membrane protein transport	0.086309486	MACF1, ANK3	21.82196231	0.879618131
GO:0051225~spindle assembly	0.086309486	INO80, NCOR1	21.82196231	0.879618131
GO:0000281~mitotic cytokinesis	0.09240346	ANK3, APC	20.3169994	0.886411378
GO:0043967~histone H4 acetylation	0.09845751	BRCA2, EP400	19.00622524	0.892303076
GO:0071479~cellular response to ionizing radiation	0.09845751	TP53, INO80	19.00622524	0.892303076
GO:0042771~intrinsic apoptotic signaling pathway in response to DNA damage by p53 class mediator	0.09845751	TP53, BRCA2	19.00622524	0.892303076
GO:0010332~response to gamma radiation	0.09845751	TP53, BRCA2	19.00622524	0.892303076

Supplementary Table 4: Significantly mutated genes between BGI APOBEC-high and APOBEC-low groups

Hugo_symbol	BGI APOBEC high	BGI APOBEC low	*P* Value	or	ci.up	ci.low
PIK3CA	22	2	0.009442226	6.456114212	1.407854985	60.85494078
ERBB2	10	0	0.029619109	Inf	1.046511182	Inf
TP53	24	4	0.0315225	3.428082331	1.016038104	15.08757306
ARID1A	14	1	0.033945669	7.278853235	1.009300159	322.2380714

Supplementary Table 5: Gene ontology biological processes for genes highly expressed in TCGA APOBEC-high tumors. See Supplementary_Table_5

Supplementary Table 6: Gene ontology biological processes for genes highly expressed in TCGA APOBEC-low tumors

Term	*P* Value	Genes	Fold enrichment	Benjamini
GO:0006412~translation	3.02E–18	MRPS36, RPL19, RPL14, SNU13, RPS2, RPL23, RPL6, RPS14, RPL8, RPL3, RPL5, RPL4, RSL24D1, RPL7A, RPL12, RPL10A, RMND1, RPS23, GATB	18.82178043	1.12E–15
GO:0019083~viral transcription	8.48E–18	RPL19, RPL14, RPS2, AAAS, RPL23, RPL6, RPS14, RPL8, RPL3, RPL5, RPL4, RPL7A, RPL12, RPL10A, RPS23	33.56609808	1.56E–15
GO:0000184~nuclear-transcribed mRNA catabolic process, nonsense-mediated decay	2.04E–17	RPL19, RPL14, RPS2, RPL23, RPL6, EIF3E, RPS14, RPL8, RPL3, RPL5, RPL4, RPL7A, RPL12, RPL10A, RPS23	31.59162172	2.51E–15
GO:0006614~SRP-dependent cotranslational protein targeting to membrane	3.53E–17	RPL19, RPL14, RPS2, RPL23, RPL6, RPS14, RPL8, RPL3, RPL5, RPL4, RPL7A, RPL12, RPL10A, RPS23	37.32740553	3.26E–15
GO:0006364~rRNA processing	1.30E–16	RPL19, RPL14, SNU13, RPS2, RPL23, RPL6, RPS14, RPL8, RPL3, YBEY, RPL5, RPL4, RPL7A, RPL12, RPL10A, MTERF4, RPS23	19.90961082	8.22E–15
GO:0006413~translational initiation	1.55E–16	RPL19, RPL14, RPS2, RPL23, RPL6, EIF3E, RPS14, RPL8, RPL3, RPL5, RPL4, RPL7A, RPL12, RPL10A, RPS23	27.4408977	6.88E–15
GO:0000027~ribosomal large subunit assembly	1.24E–06	RPL6, RPL3, RPL5, RSL24D1, RPL12	59.67306326	6.53E–05
GO:0042254~ribosome biogenesis	3.77E–04	ZNF658, SNU13, RSL24D1, RPL7A	27.84742952	0.017260352
GO:0000470~maturation of LSU-rRNA	0.001545702	SNU13, RPL10A, RPL7A	50.12537313	0.061453456
GO:0032981~mitochondrial respiratory chain complex I assembly	0.001946218	OXA1L, NDUFA7, BCS1L, NDUFA10	15.91281687	0.06936246
GO:0055114~oxidation-reduction process	0.008418884	MRPS36, OXA1L, GMPR2, HSD17B2, ALDH4A1, NDUFA10, TECR, APEX1	3.386849536	0.246940332
GO:0032543~mitochondrial translation	0.008794664	NDUFA7, MTERF4, GATB	20.88557214	0.237865423
GO:0098609~cell-cell adhesion	0.021900275	RPL14, RPL6, EIF3E, RPL7A, RPS2	4.624111913	0.466629445
GO:0030490~maturation of SSU-rRNA	0.053662512	RPS14, SNU13	35.80383795	0.766308903
GO:0071353~cellular response to interleukin-4	0.09024634	ADAMTS13, RPL3	20.88557214	0.902382931
GO:0042273~ribosomal large subunit biogenesis	0.093827193	RPL14, RPL5	20.05014925	0.896918845
GO:0002181~cytoplasmic translation	0.093827193	RPL6, RPL8	20.05014925	0.896918845
GO:0006506~GPI anchor biosynthetic process	0.097394164	PIGZ, PIGH	19.27898967	0.891844701

Supplementary Table 7: Gene ontology biological processes for genes positively correlated with APOBEC enrichment score in the TCGA dataset

Term	*P* Value	Genes	Fold enrichment	Benjamini
GO:0060333~interferon-gamma-mediated signaling pathway	1.08E−12	NMI, FCGR1A, FCGR1B, IRF1, IFI30, HLA-DPA1, HLA-E, STAT1, HLA-DRA, GBP1, B2M	32.51971831	5.80E–10
GO:0050776~regulation of immune response	1.50E–07	FCGR1A, SLA2, IRF1, NECTIN2, FCGR3A, HLA-E, KLRD1, CD226, KIR2DL4, B2M	11.79213483	4.03E–05
GO:0006955~immune response	5.92E–07	IL7, FASLG, SAMHD1, CTSS, HLA-E, HLA-DMA, CCL4, B2M, FCGR1A, FCGR1B, HLA-DPA1, FCGR3A, HLA-DRA	6.481472684	1.06E–04
GO:0019886~antigen processing and presentation of exogenous peptide antigen via MHC class II	7.24E–05	IFI30, FCER1G, HLA-DPA1, CTSS, HLA-DMA, HLA-DRA	13.68913043	0.009676253
GO:0008037~cell recognition	1.45E–04	TIGIT, NECTIN2, CLEC7A, CD226	38.16363636	0.015422393
GO:0002250~adaptive immune response	6.63E–04	HAVCR2, BTN3A1, FCGR1B, TAP1, CTSS, HLA-E	8.509459459	0.057657338
GO:0019882~antigen processing and presentation	0.002205061	HLA-DPA1, CTSS, HLA-E, HLA-DRA	15.26545455	0.155783637
GO:0002504~antigen processing and presentation of peptide or polysaccharide antigen via MHC class II	0.002839106	HLA-DPA1, HLA-DMA, HLA-DRA	37.04117647	0.173740221
GO:0002479~antigen processing and presentation of exogenous peptide antigen via MHC class I, TAP-dependent	0.003249051	FCGR1A, FCGR1B, HLA-E, B2M	13.32698413	0.176486754
GO:0060337~type I interferon signaling pathway	0.003397396	IRF1, SAMHD1, HLA-E, STAT1	13.11875	0.16702428
GO:0045087~innate immune response	0.004086313	HAVCR2, NLRC5, FCER1G, CLEC7A, C2, HLA-E, KLRD1, B2M	3.905116279	0.181183331
GO:0050830~defense response to Gram-positive bacterium	0.007505976	HAVCR2, DROSHA, HLA-E, B2M	9.877647059	0.28620492
GO:0051607~defense response to virus	0.007627996	NLRC5, IRF1, SAMHD1, STAT1, GBP1	6.360606061	0.271161301
GO:0002474~antigen processing and presentation of peptide antigen via MHC class I	0.00872908	TAP1, HLA-E, B2M	20.99	0.285585872
GO:0042590~antigen processing and presentation of exogenous peptide antigen via MHC class I	0.009387388	IFI30, FCER1G	209.9	0.28655921
GO:0033005~positive regulation of mast cell activation	0.009387388	NECTIN2, CD226	209.9	0.28655921
GO:0006911~phagocytosis, engulfment	0.011760224	FCGR1A, VAMP7, FCER1G	17.99142857	0.327694048
GO:0002891~positive regulation of immunoglobulin mediated immune response	0.0140484	NECTIN2, CD226	139.9333333	0.360399019
GO:0032729~positive regulation of interferon-gamma production	0.01978803	HAVCR2, HLA-DPA1, CD226	13.68913043	0.449133058
GO:0007157~heterophilic cell-cell adhesion via plasma membrane cell adhesion molecules	0.023139967	TIGIT, NECTIN2, CD226	12.594	0.484023583
GO:0002503~peptide antigen assembly with MHC class II protein complex	0.02330556	HLA-DMA, HLA-DRA	83.96	0.469089267
GO:0002481~antigen processing and presentation of exogenous protein antigen via MHC class Ib, TAP-dependent	0.032476894	TAP1, B2M	59.97142857	0.570127053
GO:0002860~positive regulation of natural killer cell mediated cytotoxicity directed against tumor cell target	0.032476894	NECTIN2, CD226	59.97142857	0.570127053
GO:0019885~antigen processing and presentation of endogenous peptide antigen via MHC class I	0.032476894	TAP1, B2M	59.97142857	0.570127053
GO:0072643~interferon-gamma secretion	0.032476894	BTN3A1, BTN3A2	59.97142857	0.570127053
GO:0006954~inflammatory response	0.033048752	HAVCR2, APOL3, NMI, CXCR6, CLEC7A, CCL4	3.322955145	0.559709794
GO:0002456~T cell mediated immunity	0.037030622	BTN3A3, BTN3A2	52.475	0.585631286
GO:0002480~antigen processing and presentation of exogenous peptide antigen via MHC class I, TAP-independent	0.041563188	HLA-E, B2M	46.64444444	0.613203089
GO:0007165~signal transduction	0.045115467	APOL3, IL2RB, KLRC3, FCGR1A, SOS1, NECTIN2, FASLG, M6PR, CCL4, CD226, KIR2DL4	1.988716624	0.629025165
GO:0048280~vesicle fusion with Golgi apparatus	0.046074688	VAMP7, VTI1A	41.98	0.622520819
GO:0045088~regulation of innate immune response	0.059483761	IRF1, SAMHD1	32.29230769	0.704685161
GO:0001916~positive regulation of T cell mediated cytotoxicity	0.059483761	HLA-E, B2M	32.29230769	0.704685161
GO:0035458~cellular response to interferon-beta	0.072706699	IRF1, STAT1	26.2375	0.764889925
GO:0050870~positive regulation of T cell activation	0.081419844	CD47, HLA-DPA1	23.32222222	0.792494465
GO:0045954~positive regulation of natural killer cell mediated cytotoxicity	0.081419844	NECTIN2, CD226	23.32222222	0.792494465
GO:0032753~positive regulation of interleukin-4 production	0.090052144	HAVCR2, HLA-E	20.99	0.815330377

Supplementary Table 8: Gene ontology biological processes for genes negatively correlated with APOBEC enrichment score in the TCGA dataset

Term	*P* Value	Genes	Fold enrichment	Benjamini
GO:0006413~translational initiation	2.24E–30	RPL17, RPL19, RPL14, RPL26, RPS4X, RPS2, EIF4B, RPS18, RPL7, RPL23, RPS3A, RPS14, EIF3E, RPL8, RPL3, RPS13, EIF3L, RPL5, RPL11, RPL4, RPL10A, RPL7A, RPL12, RPS23	37.71364402	8.90E–28
GO:0019083~viral transcription	8.16E–29	RPL17, RPL19, RPL14, RPL26, RPS4X, RPS2, RPS18, AAAS, RPL7, RPL23, RPS3A, RPS14, RPL8, RPL3, RPS13, RPL5, RPL11, RPL4, RPL7A, RPL12, RPL10A, RPS23	42.28754579	1.62E–26
GO:0006614~SRP-dependent cotranslational protein targeting to membrane	1.20E–28	RPL17, RPL19, RPL14, RPL26, RPS4X, RPS2, RPS18, RPL7, RPL23, RPS3A, RPS14, RPL8, RPL3, RPS13, RPL5, RPL11, RPL4, RPL7A, RPL12, RPL10A, RPS23	48.09492635	1.59E–26
GO:0000184~nuclear-transcribed mRNA catabolic process, nonsense-mediated decay	3.23E–28	RPL17, RPL19, RPL14, RPL26, RPS4X, RPS2, RPS18, RPL7, RPL23, RPS3A, RPS14, EIF3E, RPL8, RPL3, RPS13, RPL5, RPL11, RPL4, RPL7A, RPL12, RPL10A, RPS23	39.80004309	3.21E–26
GO:0006364~rRNA processing	3.71E–24	RPL17, RPL19, RPL14, RPL26, RPS4X, RPS2, RPS18, RPL7, RPL23, RPS3A, RPS14, RPL8, RPL3, YBEY, RPS13, RPL5, RPL11, RPL4, MTERF4, RPL7A, RPL12, RPL10A, RPS23	23.13779056	2.95E–22
GO:0006412~translation	1.55E–22	RPL17, RPL19, RPL14, RPL26, RPS4X, RPS2, RPS18, RPL7, RPL23, RPS3A, RPS14, RPL8, RPL3, RPS13, RPL5, RPL11, RPL4, RPL7A, RPL12, RPL10A, RMND1, RPS23, GATB	19.57109557	1.03E–20
GO:0042273~ribosomal large subunit biogenesis	4.80E–06	RPL14, RPL7, RPL26, RPL5, RPL11	43.05641026	2.73E–04
GO:0000027~ribosomal large subunit assembly	1.16E–04	RPL3, RPL5, RPL11, RPL12	41.00610501	0.005763748
GO:0002181~cytoplasmic translation	1.98E–04	EIF4B, RPL7, RPL8, RPL26	34.44512821	0.008731036
GO:0045471~response to ethanol	0.001376284	EEF1B2, PEMT, EEF2, RPL10A, RPS4X	10.25152625	0.053338612
GO:0000470~maturation of LSU-rRNA	0.002096572	RPL10A, RPL7A, NSA2	43.05641026	0.073125802
GO:0006414~translational elongation	0.003027919	EEF1A1, EEF1B2, EEF2	35.88034188	0.095685728
GO:0032259~methylation	0.004579898	FAM86B1, DPH5, PEMT, GAMT	11.79627678	0.131108831
GO:0001731~formation of translation preinitiation complex	0.004933281	EIF4B, EIF3E, EIF3L	28.08026756	0.131157316
GO:0098609~cell-cell adhesion	0.008107612	RACK1, RPL14, EIF3E, EEF2, RPL7A, RPS2	4.766392279	0.194263719
GO:0042254~ribosome biogenesis	0.011821407	RPS18, ZNF658, RPL7A	17.94017094	0.256070799
GO:0006446~regulation of translational initiation	0.011821407	EIF4B, EIF3E, EIF3L	17.94017094	0.256070799
GO:0046498~S-adenosylhomocysteine metabolic process	0.018217907	PEMT, GAMT	107.6410256	0.349781156
GO:0046500~S-adenosylmethionine metabolic process	0.031665991	PEMT, GAMT	61.50915751	0.509090644
GO:0017183~peptidyl-diphthamide biosynthetic process from peptidyl-histidine	0.036108155	DPH5, EEF2	53.82051282	0.537155574
GO:0045666~positive regulation of neuron differentiation	0.049829261	HOXD3, CYB5D2, BMP7	8.280078895	0.638379638
GO:0046037~GMP metabolic process	0.066645097	PDE6B, GMPR2	28.7042735	0.729407477
